# MicroRNA Profile for Diagnostic and Prognostic Biomarkers in Thyroid Cancer

**DOI:** 10.3390/cancers13040632

**Published:** 2021-02-05

**Authors:** Jong-Lyul Park, Seon-Kyu Kim, Sora Jeon, Chan-Kwon Jung, Yong-Sung Kim

**Affiliations:** 1 Genome Editing Research Center, Korea Research Institute of Bioscience and Biotechnology, Daejeon 34141, Korea; nlcguard@kribb.re.kr; 2 Personalized Genomic Medicine Research Center, Korea Research Institute of Bioscience and Biotechnology, Daejeon 34141, Korea; seonkyu@kribb.re.kr; 3 Department of Bioinformatics, University of Science and Technology, Daejeon 34141, Korea; 4 Cancer Research Institute, College of Medicine, The Catholic University of Korea, Seoul 06591, Korea; thfk38@nate.com; 5 Department of Hospital Pathology, College of Medicine, The Catholic University of Korea, Seoul 06591, Korea; 6 Department of Functional Genomics, University of Science and Technology, Daejeon 34141, Korea

**Keywords:** miRNA, thyroid neoplasms, papillary thyroid carcinoma, NIFTP, miR-136, miR-21, miR-127

## Abstract

**Simple Summary:**

Thyroid nodules are frequently detected, but the majority of these nodules are benign. Molecular markers have become useful for diagnosing the minority of thyroid cancer. We performed high-throughput small RNA sequencing in a discovery cohort and identified three miRNAs (miR-136, miR-21, and miR-127) as potential biomarkers for thyroid cancer. We validated the diagnostic and prognostic utilities of three miRNAs in patients with thyroid cancer in an independent cohort. High expression of the three miRNAs could be used to differentiate thyroid cancers from benign tumors and tumors with extremely low malignant potential. In patients with thyroid cancers, a high expression of three miRNAs was associated with poor clinicopathological features and recurrent or persistent disease following surgery. Therefore, testing for high expression levels of these three miRNAs in thyroid nodules may be useful for diagnosing and assessing the recurrence of the thyroid cancer’s risk stratification.

**Abstract:**

The challenge in managing thyroid nodules is to accurately diagnose the minority of those with malignancy. We aimed to identify diagnostic and prognostic miRNA markers for thyroid nodules. In a discovery cohort, we identified 20 candidate miRNAs to differentiate between noninvasive follicular thyroid neoplasms with papillary-like nuclear features (NIFTP) and papillary thyroid carcinomas (PTC) by using the high-throughput small RNA sequencing method. We then selected three miRNAs (miR-136, miR-21, and miR-127) that were differentially expressed between the PTC follicular variant and other variants in The Cancer Genome Atlas data. High expression of three miRNAs differentiated thyroid cancer from nonmalignant tumors, with an area under curve (AUC) of 0.76–0.81 in an independent cohort. In patients with differentiated thyroid cancer, the high-level expression of the three miRNAs was an independent indicator for both distant metastases and recurrent or persistent disease. In patients with PTC, a high expression of miRNAs was associated with an aggressive histologic variant, extrathyroidal extension, distant metastasis, or recurrent or persistent disease. Three miRNAs may be used as diagnostic markers for differentiating thyroid cancers from benign tumors and tumors with extremely low malignant potential (NIFTP), as well as prognostic markers for predicting the risk of recurrent/persistent disease for differentiated thyroid cancer.

## 1. Introduction

The prevalence of thyroid nodules varies with the detection methods. Thyroid nodules are detected in 5% of the adults on palpation [1] and 33% to 68% on ultrasound [2,3]. Although most thyroid nodules are benign, thyroid cancer is the most common endocrine malignancy. The age-standardized (world population) incidence rate of thyroid cancer available online at the Global Cancer Observatory (https://gco.iarc.fr/) is 10.2 per 100,000 females and 3.1 per 100,000 males in 2018 [4]. Papillary thyroid carcinoma (PTC) is the most common subtype, accounting for more than 80% of all thyroid cancers [5]. The incidence of thyroid cancer has been increasing over the past three decades in many countries, mainly because of an increase in the diagnosis of PTC and improvements in the detection and diagnosis of small cancers [6,7,8,9,10]. However, the mortality rate from thyroid cancers has remained stable over the same period despite the increasing incidence of thyroid cancer [4,10,11]. The age-standardized mortality rate was 0.42 per 100,000 people in 2018 [4]. The estimated overdiagnosis rates for thyroid cancers range from 50% to 90% of newly diagnosed cases in women, depending upon the regions and health care environment [4]. Therefore, to reduce overtreatment of nonfatal thyroid cancers, there is a clinical need for biomarkers to enable a more accurate diagnosis and risk assessment for thyroid cancers.

MicroRNAs (miRNAs) are the major post-transcriptional regulators of messenger RNA (mRNA) expression, leading to downregulation or upregulation of protein synthesis [12]. Deregulation of miRNA expression plays a role in the development, progression, and cell death of thyroid tumors [13,14,15,16]. From previous studies performed on PTC [15,16], the most consistently upregulated miRNAs were miR-146b, miR-181b, miR-221, and miR-222 [13]. Tumor suppressor miRNAs in PTC include miR-137 and miR-451 [13]. 

Several studies have evaluated the diagnostic and prognostic role of miRNA expression levels in patients with thyroid nodules [13,14,17]. Traditionally, the expression profiling of miRNAs has been studied by hybridization-based methods including microarrays. Recently, next-generation sequencing (NGS)-based RNA-seq has emerged as an alternative gene expression profiling technology for miRNAs. The NGS technology can cover all coding and noncoding RNAs and more accurately measure their expression level changes than can microarray platforms. Despite advances in molecular thyroid cancer diagnostics, there is a limited number of studies of NGS-based miRNA diagnostics for thyroid cancer in the literature.

In the present study, we aimed to obtain high-throughput miRNA-expression profiles and subsequently identify miRNA markers to differentiate thyroid cancers from noninvasive follicular thyroid neoplasm with papillary-like nuclear features (NIFTP) and benign thyroid nodules. Furthermore, the role of miRNA markers in the risk stratification to predict recurrence was further revealed by analyzing the association of their expression levels with clinicopathologic parameters in an independent cohort. 

## 2. Results

### 2.1. Baseline Characteristics

Fresh frozen tissue samples (*n* = 34) were used for the discovery of candidate biomarkers. The biomarker validation was performed in a larger number of formalin-fixed paraffin-embedded (FFPE) tissue samples (*n* = 233). The demographic and clinicopathologic features are summarized in Table 1.

### 2.2. Identification of NIFTP-Specific Differentially Expressed miRNAs

We carried out an expression profiling of miRNAs of 34 thyroid tissues including nontumor (*n* = 7), NIFTP (*n* = 6), invasive encapsulated follicular variant (IEFV) PTC (*n* = 3), classic PTC (*n* = 11), and tall cell variant (TCV) PTC (*n* = 7) in the discovery cohort. 

**Figure 1:**
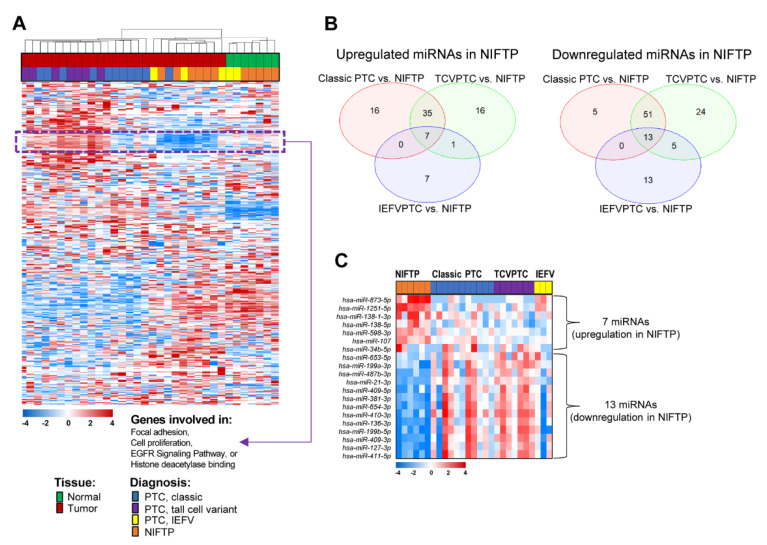
The miRNA expression profiling of thyroid tumors in a discovery cohort (*n* = 34). (**A**) The miRNA expression profiling by unsupervised hierarchical cluster analysis. A total of 712 miRNAs were selected for a cluster analysis (standard deviation > 0.7). A group of noninvasive follicular thyroid neoplasm with papillary-like nuclear features (NIFTP) and invasive encapsulated follicular variant (IEFV) of papillary thyroid carcinoma (PTC) was differentiated from a group of classic PTC and tall cell variant (TCV) by a subset of 62 miRNAs highlighted by purple dashed lines. (**B**) Comparisons of differentially expressed miRNAs between NIFTP and three variants of PTC revealed seven upregulated miRNAs and 13 downregulated miRNAs in NIFTP as compared to PTCs. (**C**) A heatmap of differentially expressed miRNAs in the thyroid tumor subgroup.

By applying unsupervised hierarchical clustering with a total of 712 miRNAs showing varying expression changes across thyroid samples (standard deviation (SD) > 0.7), we found that thyroid tumor samples were well differentiated from normal thyroid tissues. When the histological data was compared among the tumor samples, those with NIFTP or IEFVPTC were found to be differently clustered from those with classic PTC or TCVPTC (Figure 1A). 

By exploring the expression patterns, we identified a distinct subset of 62 miRNAs that showed significantly different expression patterns between patients with NIFTP/IEFV and classic PTC/TCVPTC (Figure 1A). These miRNA expression levels were higher in patients with classic PTC/TCVPTC than in those with other histologic types. Among these 62 miRNAs, many of them that were involved in focal adhesion, cell proliferation, epidermal growth factor receptor (EGFR) signaling pathways, or histone deacetylase binding were significantly enriched (Figure 1A). 

To identify subsets of miRNAs exclusive to NIFTP, we analyzed differentially expressed miRNAs between the classic PTC and NIFTP, TCVPTC and NIFTP, or IEFVPTC and NIFTP patient subgroups by using Venn diagrams (Figure 1B). Seven miRNAs were significantly upregulated in the NIFTP, whereas 13 miRNAs were significantly downregulated in the NIFTP (Figure 1C; Table 2).

### 2.3. Validation of Potential miRNA Markers in TCGA Data

**Figure 2:**
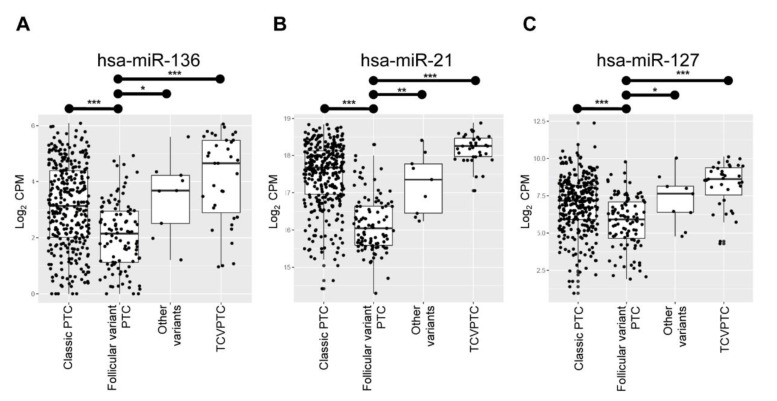
Comparison of the expression levels of miRNAs between different histological variants of papillary thyroid carcinoma (PTC) in the TCGA cohort (*n* = 466). Expression levels of hsa-miR-136 (**A**), hsa-miR-21 (**B**), and has-miR-127 (**C**) in the follicular variant were significantly lower than in the classic PTC, tall cell variant (TCV) or other histological variants. *P*-values were obtained by 2-sample *t*-tests. CPM: counts per million mapped reads. *, *P* < 0.05; **, *P* < 0.01; ***, *P* < 0.001.

Using The Cancer Genome Atlas (TCGA) data set, we sought to verify these twenty miRNAs that were differentially expressed in the NIFTP of the original cohort. Among the candidates, three miRNAs (i.e., miR-136, miR-21, and miR-127) showed significant differences in expression levels between the follicular variant and the others (i.e., classic PTC, TCVPTC, or other variants) as shown in Figure 2, consistent with the results in the discovery cohort. These three miRNAs were applied into the next further downstream experimental validations.

### 2.4. Validation of Potential miRNA Markers in an Independent Cohort

The integrity of total RNA isolated from FFPE samples showed an RNA integrity number (RIN) ranging 2.0 to 3.0. Because expression of miRNA is highly stable, even in degraded human tissue RNA and cell samples [18,19,20], we used the RNA samples extracted from 233 FFPE tissue samples for the miRNA marker validation study by using a TaqMan-based qRT-PCR assay. 

**Figure 3:**
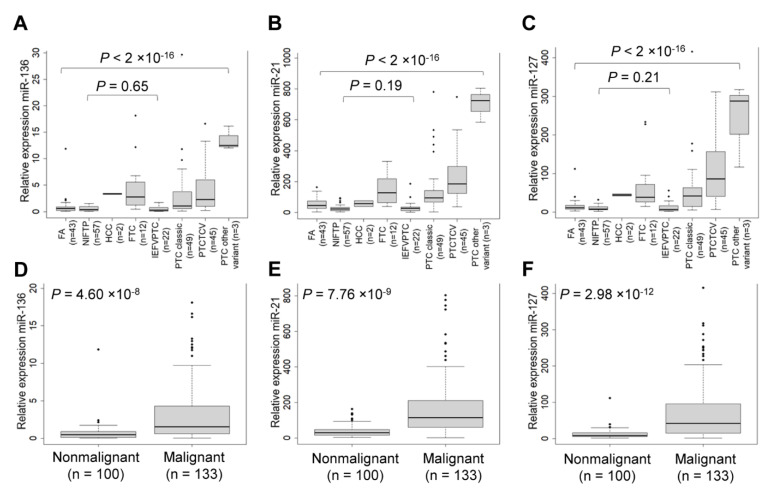
Evaluation of selected miRNA candidate in an independent cohort (*n* = 233) including follicular adenoma (FA, *n* = 43), noninvasive follicular thyroid neoplasm with papillary-like nuclear features (NIFTP, *n* = 57), Hürthle cell carcinoma (*n* = 2), follicular thyroid carcinoma (FTC, *n* = 12), invasive encapsulated follicular variant of papillary thyroid carcinoma (IEFVPTC, *n* = 22), classic papillary thyroid carcinoma (PTC, *n* = 49), PTC tall cell variant (TCV, *n* = 45), and PTC other variant (*n* = 3). The expression levels of miR-137 (**A**), miR-21 (**B**), and miR-127 (**C**) were compared between the different tumor types. All samples were further grouped into 100 non-malignant (FA and NIFTP) and 133 malignant (HCC, FTC, and PTC) tumors. The expression levels of miR-137 (**D**), miR-21 (**E**), and miR-127 (**F**) are significantly higher in malignant tumors than in non-malignant tumors. U6 snRNA was used as a reference gene to normalize miRNA results. The relative miRNA expression was calculated using the following formula: 2^(U6_ct_ − target miRNA_ct_) + 15. Ct, cycle threshold.

We observed that expression of three candidate miRNAs was more highly expressed in PTC than in other thyroid tumors (Figure 3A–C). The three selected miRNA markers were further evaluated for their capacity to differentiate nonmalignant tumors, including follicular adenoma (FA) and NIFTP from malignant tumors, including PTC, follicular thyroid carcinoma (FTC), and Hürthle cell carcinoma (Figure 3D–F). 

**Figure 4:**
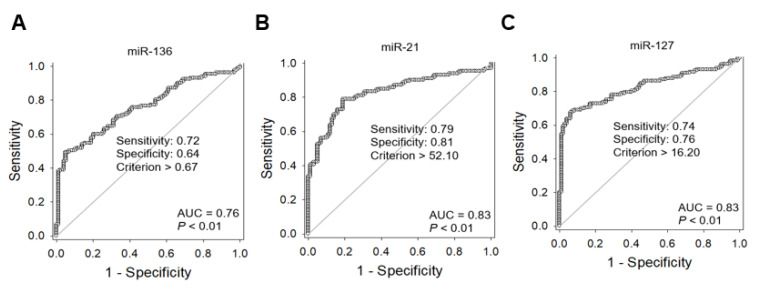
Receiver operating characteristic curve analysis of three selected miRNA markers for discrimination of nonmalignant tumors (follicular adenoma and NIFTP) from papillary thyroid carcinoma, follicular thyroid carcinoma, and Hürthle cell carcinoma. The area under the curve (AUC) indicates the probability that the classifier ranks a randomly chosen positive instance higher than a randomly chosen negative instance. The criteria for high miRNA expression levels of miR-136 (**A**), miR-21 (**B**), and miR-127 (**C**) were defined using the AUC.

To estimate the area under the curve (AUC) value, we performed receiver operating characteristic (ROC) analysis. The AUC values of miR-136, miR-21, and miR-127 were 0.76, 0.83, and 0.83, respectively (Figure 4A–C). Their optimal cutoff values and sensitivity and specificity for each marker of the diagnosis of malignancy are described in Figure 3.

### 2.5. Clinicopathologic Utility of the Three miRNA Markers in Thyroid Cancer

The expression levels of three miRNAs were classified into two sub-groups of high-or low-expression based on their median expression values. We then categorized all patients into four groups based on the number of markers showing high miRNA expression levels: all low (group 1, *n* = 88), one high (group 2, *n* = 24), two high (group 3, *n* = 28), or all high (group 4, *n* = 93). 

**Figure 5:**
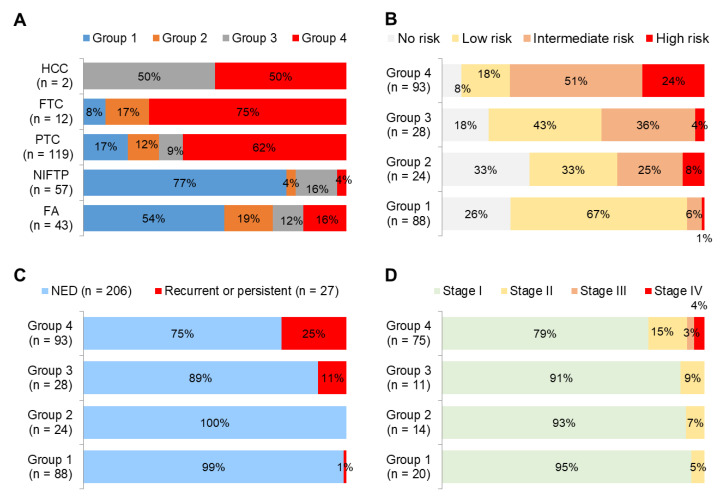
Diagnostic performance from the combination of three miRNA (miR-136, miR-21, and miR-127) markers. All thyroid tumors (*n* = 233) were divided into an all low (group 1, *n* = 88), one high (group 2, *n* = 24), two high (group 3, *n* = 28), and all high (group 4, *n* = 93) levels with three miRNA markers. The distribution of thyroid tumors (**A**), American Thyroid Association recurrence risk (**B**), and recurrent or persistent disease (**C**) in all 233 thyroid tumors, and cancer stage (**D**) in 120 thyroid cancers were stratified based on these four groups. Hürthle cell carcinoma; FTC, follicular thyroid carcinoma; PTC, papillary thyroid carcinoma; NIFTP, noninvasive follicular thyroid neoplasm with papillary-like nuclear features; FA, follicular adenoma; NED, no evidence of disease.

Benign tumors (FA) and tumors with extremely low malignant potential (NIFTP) were mostly found in group 1 whereas malignant tumors (PTC, Hürthle cell carcinoma, and FTC) were mostly found in group 4 (Figure 5A). Patients classified as high-risk according to the American Thyroid Association (ATA) recurrence risk stratification system were most frequently observed in group 4 (Figure 5B). Recurrent or persistent diseases were mostly found in group 4 (Figure 5C). Stage III and IV were only found in group 4 (Figure 5D).

**Figure 6:**
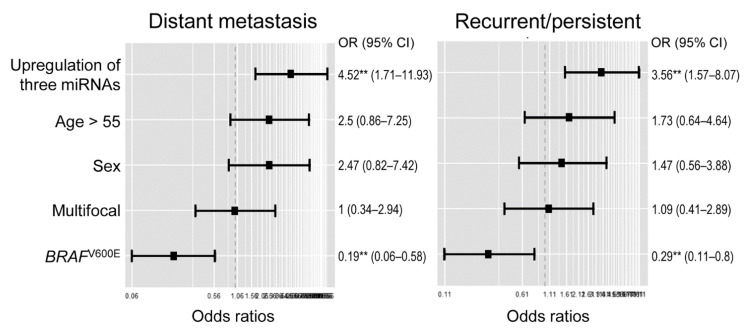
Multivariate generalized linear model analyses for risk factors associated with adverse outcomes in 133 patients with differentiated thyroid cancer (papillary thyroid carcinoma, follicular thyroid carcinoma, and Hürthle cell carcinoma). Three miRNA markers consist of miR-136, miR-21, and miR-127. **, *P* < 0.005

Multivariate generalized linear model analysis showed that high expression levels of three miRNA markers were significant predictors for recurrent or persistent disease (odds ratio, 3.56; 95% confidence interval [CI], 1.57–8.07) and distant metastases (odds ratio, 4.52; 95% CI, 1.71–11.93) in 133 patients with differentiated thyroid cancer (Figure 6).

### 2.6. Clinicopathologic Utility of the Three miRNA Markers in Patients with PTC

A subgroup analysis was conducted in 119 patients with PTC, as shown in Table 3. High expression levels of miRNAs (miR-136, miR-21, and miR-127) were associated with histologic variants (*P* < 0.001), extrathyroidal extension (*P* = 0.003), distant metastases (*P* = 0.038), recurrent or persistent disease (*P* = 0.013), and increased ATA recurrence risk (*P* < 0.001). In addition, expression levels of miR-21 were associated with lymph node metastasis and *BRAF*^V600E^ mutation. 

## 3. Discussion

Many miRNAs have been investigated as biomarkers for thyroid cancer, and their number is continuously increasing. The main role of these miRNAs is the regulation of cancer-related signaling pathways involved in cell proliferation, survival, adhesion, invasion, and migration [14]. Generally, oncomiRs and tumor suppressor miRNAs are overexpressed and underexpressed in thyroid cancer, respectively. Group profiling strategies have been used to identify the dysregulation of miRNAs in thyroid cancer. However, there has been inconsistent miRNA profiling data in different studies, depending on the measurement methods and the algorithms necessary to associate the miRNAs. We performed small RNA-seq analysis to detect both novel and known miRNAs to differentiate benign tumors (FA) and tumors with extremely low malignant potential (NIFTP) from malignant thyroid tumors. Three candidate miRNAs were validated for diagnostic and prognostic utility in an independent cohort by TaqMan-based qRT-PCR assay. 

NIFTP is a borderline thyroid tumor that has an indolent clinical outcome and overlapping histologic and molecular features with FA and with IEFVPTC [5]. It is difficult to distinguish these follicular-patterned tumors by clinical and cytologic examination before surgery. Molecular analysis cannot accurately differentiate these tumors because NIFTPs have molecular profiles similar to those of FA and IEFVPTC, with frequent *RAS*-like mutations [21,22,23]. However, recent studies have shown the miRNA expression profiles of NIFTPs to be different from those of other thyroid tumors [24,25]. 

In our study, we first identified NIFTP-specific miRNAs to differentiate malignant and borderline (NIFTP) tumors from benign tumors and to improve the diagnostic accuracy of miRNA markers for the differential diagnosis of benign and malignant tumors. In the discovery cohort, 20 miRNAs were found to be differentially expressed between NIFTP and other thyroid tumors. Of these, seven were upregulated (miR-873-5p, miR-1251-5p, miR-138-1-3p, miR-138-5p, miR-598-3p, miR-107, and miR-34b-5p) and 13 (miR-653-5p, miR-199a-3p, miR-487b-3p, miR-21-3p, miR-409-5p, miR-381-3p, miR-654-3p, miR-410-3p, miR-136-3p, miR-199b-5p, miR-409-3p, miR-127-3p, and miR-411-5p) were downregulated. As a next step, we evaluated whether 20 candidate miRNAs are differentially expressed between the follicular variant of PTC and other PTC variants in the PTC cohort of the TCGA data set. Finally, we selected three miRNA markers (miR-21, miR-136, and miR-127) for their clinical utility in thyroid tumors. 

We showed that the expression of three miRNA markers was higher in differentiated thyroid cancers than nonmalignant tumors (FA and NIFTP). A high-expression level of these miRNAs was an independent prognostic indicator for both distant metastases and recurrent or persistent disease in patients with differentiated thyroid cancers. Most cases of FA and NIFTP had low expression levels of three miRNA markers. In patients with PTC, a high-expression of miRNAs was associated with aggressive histologic variants, extrathyroidal extensions, distant metastases, recurrent or persistent disease, and a high recurrence risk as defined by the ATA. 

Growing evidence shows that miR-21 plays an oncogenic role in cancer development and progression and is a predictive biomarker of poor prognosis in hematologic and solid cancers [26,27]. The upregulation of miR-21 has also been reported in thyroid cancers, including PTC, FTC, and medullary thyroid carcinoma. It is correlated with tumor aggressiveness and advanced cancer stage [13,17,28,29]. The expression of miR-21 is regulated by DNA methylation in PTC [30]. Target genes of miR-21 include phosphatase and tensin homolog (*PTEN*), thyroid hormone receptor beta (*THRB),* programmed cell death 4 (*PDCD4),* B-cell lymphoma 2 (*BCL2*), and cell division cycle 25A (*CDC25A*) genes [13]. A recent study showed that the overexpression of miR-21-3p and miR-21-5p in FTC and FA downregulated the expression of tumor suppressor genes, such as metalloproteinase-3 (*TIMP3*), methionine adenosyltransferase 2 (*MAT2A*), transforming growth factor beta receptor II (*TGFBR2*), and plasminogen activator (*PLAT*) genes [29].

Although this is an extremely limited study, the expression of miR-136-5p was higher in thyroid neoplasms, especially in invasive PTC with lymph node metastasis than it was in nontumor tissues [31]. In the TCGA data set, a high expression of miR-136-5p was associated with older age (>50 years), high pathologic T category, lymph node metastasis, and advanced cancer stage [32]. However, contradictory studies exist concerning whether miR-136 is an oncogene or a tumor suppressor. The expression of miR-136 acts as an oncogene in lung cancer [33] and gastric cancer [34]. A previous study suggested that the expression of miR-136 downregulates the PTEN expression and subsequent activation of AKT expression in gastric cancer cells [34]. However, other studies reported miR-136 plays a tumor suppressor role in glioma [35], triple-negative breast cancer [36], colon cancer [37], and gallbladder cancer [38]. A previous study reported that miR-136 suppressed the epithelial-to-mesenchymal transition of cancer cells by regulating the RAS protein activator like 2 (*RASAL2*) gene in triple-negative breast cancer [36]. Another study done on colon cancer reported that miR-136 suppressed the epithelial-to-mesenchymal transition of cancer cells by targeting the migration and invasion enhancer 1 (*MIEN1*) gene. Its expression level in colon cancer tissue was inversely correlated with tumor size, lymph node metastasis, and cancer stage [37]. In the gallbladder, overexpression of miR-136 suppressed cancer cell proliferation and enhanced apoptosis of cancer cells via inhibition of mitogen-activated protein kinase 4 (*MAP2K4*) gene expression [38]. 

As with miR-136, the miR-127 plays a dual role as an oncogene and a tumor suppressor, depending upon the cancer tissue type involved. In the TCGA data set, the miR-127 expression level was higher in stage III PTC than in stage II tumors [39]. In medullary thyroid cancer, miR-127 was more frequently upregulated in *RET* wild-type cancers [40]. In our study, the high expression of miR-127 was associated with thyroid cancers and a predictive marker for tumor recurrence. In contrast, miR-127 has also been reported to act as a tumor suppressor in various tumor types. The target genes downregulated by miR-127 have been shown as follows: B-cell lymphoma 6 (*BCL6*) in breast cancer [41], replication initiator 1 (*REPIN1*) in glioma [42], formin-like 3 (*FMNL3*) in esophageal cancer [43], cytochrome C oxidase assembly factor 1 homologue (*COA1*) in giant cell tumor of bone [44], and delta like non-canonical notch ligand 1 (*DLK1*) in melanoma [45]. 

Therefore, miR-136 and miR-127 play different roles depending on the cancer types and microenvironment. Further studies are required to reveal the oncogenic role of miR-136 and miR-127 in thyroid cancers. 

Intracellular and extracellular miRNAs are relatively stable in clinical tissues, blood, and body fluids under various conditions [46,47,48]. These characteristics enable reliable detection of common miRNAs in different types of cytology and pathology specimens. Currently, two miRNA-based molecular tests are commercially available for indeterminate thyroid nodules on fine needle aspiration cytology [49]: ThyraMIR (Interpace Diagnostics, Parsippany, NJ, USA) using a panel of 10 miRNAs and RosettaGX Reveal (Rosetta Genomics, Philadelphia, PA, USA) using a panel of 24 miRNAs. The miRNA-base tests classify cytologically indeterminate thyroid nodules into benign or suspicious for malignancy by miRNA profiling. This practice highlights the clinical relevance of miRNA tests in the diagnosis of thyroid nodules. As the miRNA panels in both tests do not include our three miRNA markers, the diagnostic performance of miRNA testing may increase by incorporating our three miRNAs. However, further studies are needed to validate the clinical utility of miRNA markers on cytology samples. 

## 4. Materials and Methods 

### 4.1. Study Subjects

This study was approved by the Institute Review board of Seoul St. Mary’s Hospital of the Catholic University of Korea (KC16SISI0709), and informed consent was obtained. Thyroid tumor tissue and nontumor tissues samples were obtained from the Biobank of Seoul St. Mary’s Hospital.

Detailed demographic and baseline characteristics of patients are summarized in the Table 1. The present study was performed on the same cohort and tissue samples used in our previous study investigating DNA methylation biomarkers [50].

For profiling of miRNA expression levels, we used 34 fresh frozen tissue samples, including nontumor (*n* = 7), NIFTP (*n* = 6), invasive EFVPTC (*n* = 3), classic PTC (*n* = 11), and TCVPTC (*n* = 7). 

In addition, we used 233 FFPE tissues samples to validate our selected miRNA markers by qRT-PCR. The validation cohort consisted of FA (*n* = 43), NIFTP (*n* = 57), invasive EFVPTC (*n* = 22), classic PTC (*n* = 49), TCV PTC (*n* = 45), other variants of PTC (*n* = 3), follicular thyroid carcinoma (FTC, *n* = 12), and Hürthle cell carcinoma (*n* = 2). Hürthle cell adenoma was not included within the FA group. Thyroid tumors were pathologically classified according to the 2017 World Health Organization classification of tumors of endocrine organs [5]. Cancer stages were categorized according to the 8th edition of the American Joint Committee on Cancer (AJCC) staging manual [51]. Recurrence risk was evaluated using the ATA classification for risk of recurrence [52].

### 4.2. Total RNA Isolation and Small RNA Sequencing

Total RNA was isolated from the fresh-frozen tissues and 10-μm thick paraffin-embedded tissue sections using the RecoverAll™ Total Nucleic Acid Isolation Kit (Life Technologies, Carlsbad, CA, USA), according to the manufacturer’s instructions. The quality and quantity of the extracted total RNA were analyzed with an ND-1000 spectrophotometer (Thermo Fisher Scientific, Waltham, MA, USA). In addition, the 2100 Agilent Bioanalyzer (Agilent Technologies, Waldbronn, Germany) was used for the estimation of the RNA integrity number (RIN) score. 

Small RNAs (20–30 nt) were purified from 15% Novex TBE-Urea Gel (Invitrogen) and ligated first with the 5’ RNA adaptor and then with the 3’ RNA adaptor provided by Illumina TruSeq small RNA sample preparation protocol. In each step, the ligated product was PAGE-gel purified. After first-strand synthesis and 11 cycles of PCR amplification, the product was PAGE-gel purified and submitted for sequencing on an Illumina NextSeq500 at LAS (Seoul, Korea; https://www.las.kr/).

### 4.3. Small RNA Sequencing Data Analysis

After TruSeq small RNA adapters were eliminated among the sequenced reads by Trimmomatic software (v. 0.38), the remained sequence data was mapped to the human genome (GRCh38) using bowtei2 (v. 2.3.4). The quantification was carried out using the HTSeq software package. The dataset generated by small RNA-seq is available in the National Center for Biotechnology Information (NCBI) Gene Expression Omnibus (GEO) public database under data series accession number GSE159330

### 4.4. Public miRNA-Sequencing Data Collection

Public miRNA-sequencing data of normal thyroid and PTC samples was obtained from the TCGA dataset (https://portal.gdc.cancer.gov/) to confirm the miRNA patterns of candidate miRNA markers.

### 4.5. MiRNA Expression Levels by qRT-PCR

To evaluate the miRNA expression level, we used the TaqMan MicroRNA assay (Thermo Fisher Scientific) according to manufacturer’s instructions. Briefly, 10 ng of total RNA was reverse transcribed into cDNA with each miRNA-specific primer, and then 1.44 uL of cDNA was used for the CFX96 real-time PCR system (Bio-Rad, Hercules, CA, USA). The PCR cycles were as follows: initial denaturation at 95 °C for 10 min, followed by 40 cycles at 94 °C for 15 s, and 60 °C for one minute. The expression level of each miRNA was normalized to a noncoding small nuclear U6 RNA expression that was commonly used as a reference in previous studies [53,54,55]. The relative miRNA expression level was calculated using the following formula: 2^(U6_ct_ − target miRNA_ct_) + 15.

### 4.6. BRAF Mutational Analysis

Genomic DNA was isolated from 10-μm thick paraffin-embedded tissue sections using the RecoverAll™ Total Nucleic Acid Isolation Kit (Life Technologies). After PCR amplification of the extracted DNA, the sequences of *BRAF* exon 15 were analyzed by direct sequencing of amplicons, as described previously [50,56,57].

### 4.7. Statistical Analysis

To perform miRNA expression profiling for thyroid tissue samples, a hierarchical clustering algorithm with the centered correlation coefficient as the measure of similarity and complete linkage clustering was applied. For cluster analysis, the read counts per million fragments mapped (CPM) of each sample were used to estimate the expression level of each miRNA. The CPM data was normalized by the quantile method, log_2_ transformed, and median centered across genes and samples. To estimate the significance of differences in gene expression between sample subgroups, the edgeR package, which uses a negative binomial model, was employed to detect differentially expressed miRNAs from the count data [58]. Expression differences in miRNAs were considered to be statistically significant if the *P*-value was < 0.05 and the fold difference in expression between two sample groups was ≥ 1.5. Function enrichment analysis was carried out to identify the most significant miRNA sets with miEAA software [6]. The significance of overrepresented gene sets was estimated by Fisher’s exact test. Statistical analysis was mainly carried out in the R language environment (ver. 3.5.3).

The ROC and the respective area under the ROC curve (AUC) were calculated for each miRNA marker, using the ROCR package of the R software (version 3.4.0). ROC curve analysis estimated the optimal cutoff values maximizing sensitivity and specificity between low and high levels of miRNA expression. The relationship between clinicopathologic features and the expression levels of miRNAs was analyzed using parametric (chi-squared test) and non-parametric (Fisher’s exact) assessments, where appropriate. Generalized liner model was performed to assess the associations of clinicopathologic variables and miRNA expression levels with the adverse clinical outcomes.

## 5. Conclusions

In conclusion, we report that upregulation of three miRNA markers, miR-136, miR-21 and miR-127, may serve as diagnostic and prognostic biomarker for differentiated thyroid cancer. These miRNA markers may be clinically useful for efficiently stratifying thyroid tumors.

## Figures and Tables

**Table 1:** Baseline characteristics of the subjects used in this study.

Characteristic	Fresh Frozen Samples for Discovery	FFPE Samples for Validation
Sample	34	233
Age years at diagnosis, mean (range)	44 (26–70)	47 (19–70)
Sex		
Female	17	144
Male	9	89
Tumor size (cm), mean (range)	2.1 (1.1–5.0)	2.4 (1.0–9.0)
Pathologic diagnosis		
Matched normal thyroid	7	0
Follicular adenoma	0	43
NIFTP	6	57
PTC, classic type	11	49
PTC, IEFV	3	22
PTC, tall cell variant	7	45
PTC, other variants ^1^	0	3
Follicular thyroid carcinoma ^2^	0	12
Hürthle cell carcinoma ^3^	0	2

FFPE, formalin-fixed paraffin-embedded; NIFTP, noninvasive follicular thyroid neoplasm with papillary-like nuclear features; PTC, papillary thyroid carcinoma; IEFV, invasive encapsulated follicular variant. ^1^ Other PTC variants (*n* = 3) include diffuse sclerosing (*n* = 1) and columnar cell (*n* = 2) variants. ^2^ Follicular thyroid carcinoma (*n* = 12) includes minimally invasive (*n* = 4) and encapsulated angioinvasive (*n* = 8) types. ^3^ Hürthle cell carcinoma (*n* = 2) includes minimally invasive (*n* = 1) and encapsulated angioinvasive (*n* = 1) types.

**Table 2:** Significant miRNAs that were differentially expressed in the NIFTP than in the other subgroups.

miRNAs	Expression in NIFTP	Average Fold Change (log2 Scale)	Classic PTC vs. NIFTP			TCVPTC vs. NIFTP			IEFVPTC vs. NIFTP		
			*P*-Value	Fold Change (log2 Scale)	Average CPM (log2 Scale)	*P*-Value	Fold Change (log2 Scale)	Average CPM (log2 Scale)	*p*-Value	Fold Change (log2 Scale)	Average CPM (log2 Scale)
*hsa-miR-873-5p*	UP	3.144	9.78 × 10^−10^	4.070	3.279	5.81 × 10^−8^	4.216	3.545	3.53 × 10^−2^	1.854	4.156
*Has-miR-1251-5p*	UP	2.519	1.54 × 10^−7^	2.257	6.051	2.27 × 10^−7^	2.315	6.260	4.37 × 10^−2^	1.312	6.855
*hsa-miR-138-1-3p*	UP	1.683	4.95 × 10^−3^	1.526	4.694	9.53 × 10^−4^	1.986	4.737	4.71 × 10^−2^	1.750	5.202
*hsa-miR-138-5p*	UP	1.574	1.06 × 10^−2^	1.111	6.198	4.15 × 10^−4^	1.780	6.121	2.11 × 10^−2^	1.740	6.564
*hsa-miR-598-3p*	UP	1.359	1.44 × 10^−2^	0.882	5.283	9.91 × 10^−4^	1.155	5.283	9.65 × 10^−3^	1.633	5.536
*hsa-miR-107*	UP	1.244	1.43 × 10^−2^	0.830	5.686	3.78 × 10^−4^	1.337	5.594	4.81 × 10^−2^	1.203	5.991
*hsa-miR-34b-5p*	UP	0.409	4.06 × 10^−2^	2.259	3.663	3.15 × 10^−2^	2.631	3.820	4.57 × 10^−2^	4.706	4.130
*hsa-miR-653-5p*	DOWN	−1.366	2.52 × 10^−3^	−1.653	3.500	7.87 × 10^−5^	−2.659	4.033	1.39 × 10^−2^	−2.150	3.523
*hsa-miR-199a-3p*	DOWN	−2.299	1.17 × 10^−3^	−2.443	4.204	4.21 × 10^−10^	−3.255	4.618	3.55 × 10^−2^	−1.675	3.431
*hsa-miR-487b-3p*	DOWN	−2.358	7.28 × 10^−5^	−4.258	3.175	2.64 × 10^−6^	−4.136	2.967	1.85 × 10^−2^	−3.120	2.380
*hsa-miR-21-3p*	DOWN	−2.369	1.32 × 10^−13^	−2.835	10.720	2.47 × 10^−28^	−3.289	10.919	1.97 × 10^−2^	−1.095	9.010
*hsa-miR-409-5p*	DOWN	−2.399	2.64 × 10^−4^	−2.928	3.437	4.36 × 10^−6^	−3.609	3.753	4.39 × 10^−2^	−2.176	2.756
*hsa-miR-381-3p*	DOWN	−2.542	3.61 × 10^−6^	−3.289	5.758	7.16 × 10^−15^	−3.681	5.860	6.35 × 10^−3^	−2.147	4.402
*hsa-miR-654-3p*	DOWN	−2.744	6.59 × 10^−7^	−3.864	5.413	4.32 × 10^−17^	−3.975	5.290	3.22 × 10^−3^	−2.592	3.920
*hsa-miR-410-3p*	DOWN	−3.173	1.28 × 10^−6^	−4.292	4.509	6.79 × 10^−12^	−4.411	4.428	2.33 × 10^−2^	−2.459	2.896
*hsa-miR-136-3p*	DOWN	−3.182	1.06 × 10^−7^	−4.075	5.171	3.05 × 10^−17^	−4.593	5.408	2.96 × 10^−3^	−2.651	3.601
*hsa-miR-199b-5p*	DOWN	−3.254	5.45 × 10^−8^	−3.740	8.580	1.88 × 10^−13^	−4.468	8.994	3.85 × 10^−2^	−1.832	6.468
*hsa-miR-409-3p*	DOWN	−3.623	2.02 × 10^−8^	−4.181	6.275	1.67 × 10^−21^	−4.590	6.425	7.50 × 10^−5^	−3.087	4.733
*hsa-miR-127-3p*	DOWN	−3.696	2.34 × 10^−7^	−4.035	10.032	1.78 × 10^−12^	−4.194	9.912	4.30 × 10^−3^	−2.506	8.062
*hsa-miR-411-5p*	DOWN	−3.762	4.67 × 10^−7^	−4.089	7.256	6.03 × 10^−14^	−4.271	7.181	1.63 × 10^−3^	−2.721	5.464

NIFTP, noninvasive follicular thyroid neoplasm with papillary-like nuclear features. IEFV, invasive encapsulated follicular variant. CPM, counts per million mapped reads.

**Table 3:** Relationship between clinicopathologic features and expression levels of three miRNA markers in 119 patients with papillary thyroid carcinoma.

Characteristic	miR-136		*P*-Value	miR-21		*P*-Value	miR-127		*P*-Value
	High Expression	Low Expression		High Expression	Low Expression		High Expression	Low Expression	
Age (years)			<0.001			0.289			1.000
< 55	58 (84.1%)	11 (15.9%)		69 (82.1%)	15 (17.9%)		60 (71.4%)	24 (28.6%)	
≥ 55	24 (48.0%)	26 (52.0%)		25 (71.4%)	10 (28.6%)		25 (71.4%)	10 (28.6%)	
Sex			0.362			0.404			0.044
Male	40 (74.1%)	14 (25.9%)		45 (83.3%)	9 (16.7%)		44 (81.5%)	10 (18.5%)	
Female	42 (64.6%)	23 (35.4%)		49 (75.4%)	16 (24.6%)		41 (63.1%)	24 (36.9%)	
Tumor size (cm)	2.04 ± 1.16	1.93 ± 1.11	0.645	1.95 ± 1.09	2.22 ± 1.33	0.281	2.18 ± 1.13	1.94 ± 1.15	0.312
Histologic subtypes			<0.001			<0.001			<0.001
Classic	33 (66.7%)	16 (33.3%)		43 (87.8%)	6 (12.2%)		34 (69.4%)	15 (30.6%)	
IEFV	7 (31.8%)	15 (68.2%)		5 (22.7%)	17 (77.3%)		6 (27.3%)	16 (72.7%)	
Tall cell variant	39 (86.7%)	6 (13.3%)		43 (95.6%)	2 (4.4%)		42 (93.3%)	3 (6.7%)	
Other	3 (100%)	0 (0.00%)		3 (100%)	0		3 (100%)	0	
Histologic aggressiveness			<0.001			<0.001			<0.001
Non-aggressive variant	41 (56.9%)	31 (43.1%)		49 (68.1%)	23 (31.9%)		41 (56.9%)	31 (43.1%)	
Aggressive variant	41 (87.2%)	6 (12.8%)		45 (95.7%)	2 (4.3%)		44 (93.6%)	3 (6.4%)	
Extrathyroidal extension			0.003			<0.001			<0.001
Absent	21 (50.0%)	21 (50.0%)		22 (52.4%)	20 (47.6%)		20 (47.6%)	22 (52.4%)	
Microscopic	47 (77.1%)	14 (23.0%)		56 (91.8%)	5 (8.2%)		51 (83.6%)	10 (16.4%)	
Gross	14 (87.5%)	2 (12.5%)		16 (100%)	0		14 (87.5%)	2 (12.5%)	
Multifocality			0.503			0.097			0.404
Absent	39 (65.0%)	21 (35.0%)		44 (72.1%)	17 (27.9%)		41 (67.2%)	20 (32.8%)	
Present	42 (72.4%)	16 (27.6%)		50 (86.2%)	8 (13.8%)		44 (75.9%)	14 (24.1%)	
Lymph node metastasis			0.189			<0.001			0.053
Absent	30 (61.2%)	19 (38.8%)		31 (59.6%)	21 (40.4%)		29 (60.4%)	19 (39.6%)	
Present	52 (74.3%)	18 (25.7%)		63 (94.0%)	4 (6.0%)		55 (78.6%)	15 (21.4%)	
pT category			0.637			0.583			0.148
pT1	46 (64.8%)	25 (35.2%)		56 (78.9%)	15 (21.1%)		53 (74.6%)	18 (25.4%)	
pT2	21 (72.4%)	8 (27.6%)		21 (72.4%)	8 (27.6%)		16 (55.2%)	13 (44.8%)	
pT3	10 (76.9%)	3 (23.1%)		11 (84.6%)	2 (15.4%)		11 (84.6%)	2 (15.4%)	
pT4	5 (83.3%)	1 (16.7%)		6 (100%)	0		5 (83.3%)	1 (16.67%)	
Distant metastasis			0.005			0.038			0.005
Absent	67 (64.42%)	37 (35.58%)		79 (76.0%)	25 (24.0%)		69 (67.0%)	34 (33.0%)	
Present ^1^	15 (100%)	0 (0.00%)		15 (100%)	0		15 (100%)	0	
*BRAF*^V600E^ mutation			0.615			<0.001			<0.001
Negative	87 (82.08%)	19 (17.92%)		18 (48.7%)	19 (51.4%)		16 (43.2%)	21 (56.8%)	
Positive	64 (78.05%)	18 (21.95%)		76 (92.7%)	6 (7.3%)		69 (84.0%)	13 (16.1%)	
Recurrent or persistent disease			<0.001			0.012			0.013
Absent	63 (63.00%)	37 (37.00%)		75 (75.0%)	25 (25.0%)		67 (77.0%)	33 (33.0%)	
Present	19 (100%)	0 (0.00%)		19 (100%)	0		18 (94.7)	1 (5.3%)	
ATA recurrence risk			<0.001			<0.001			<0.001
Low	14 (43.75%)	18 (56.25%)		13 (40.6%)	19 (59.4%)		14 (43.8%)	18 (56.3%)	
Intermediate	51 (75.00%)	17 (25.00%)		22 (78.6%)	6 (21.4%)		54 (79.4%)	14 (20.6%)	
High	17 (89.47%)	2 (10.53%)		19 (100%)	0		17 (89.5%)	2 (10.5%)	
AJCC stage, 8th edition			0.525			0.492			0.375
I	66 (66.00%)	34 (34.00%)		76 (76.0%)	24 (24.0%)		68 (68.0%)	32 (32.0%)	
II	11 (78.57%)	3 (21.43%)		13 (92.9%)	1 (7.1%)		12 (85.7%)	2 (14.3%)	
III	2 (100%)	0 (0.00%)		2 (100%)	0		2 (100%)	0	
IV	3 (100%)	0 (0.00%)		3 (100%)	0		3 (100%)	0	

IEFV, invasive encapsulated follicular variant; ATA, American Thyroid Association; AJCC, American Joint Committee on Cancer. ^1^ Distant metastasis includes 10 synchronous and 8 metachronous metastases.

## Data Availability

The raw data generated by small RNA-seq are available online at https://www.ncbi.nlm.nih.gov/geo/query/acc.cgi?acc=GSE159330.
